# Gynecology and women’s health care during the COVID-19 pandemic: Patient safety in surgery and prevention

**DOI:** 10.6061/clinics/2020/e2063

**Published:** 2020-06-16

**Authors:** José Maria Soares-Júnior, Isabel C.E. Sorpreso, Eduardo Vieira Motta, Edivaldo Massazo Utiyama, Edmund Chada Baracat

**Affiliations:** I Disciplina de Ginecologia, Departamento de Obstetricia e Ginecologia, Hospital das Clínicas HCFMUSP, Faculdade de Medicina, Universidade de Sao Paulo, Sao Paulo, SP, BR; II Disciplina de Cirurgia Geral e Trauma, Departamento de Cirurgia, Hospital das Clínicas HCFMUSP, Faculdade de Medicina, Universidade de Sao Paulo, Sao Paulo, SP, BR

## INTRODUCTION

In December 2019, the novel severe acute respiratory syndrome coronavirus emerged in Wuhan, China. It has since spread around the world (1,2), leading the World Health Organization (WHO) to declare a global pandemic (3). The disease caused by the virus, coronavirus disease (COVID-19), has been detected in more than 2,400,000 people worldwide and has caused more than 160,000 deaths (3-5). In Brazil, the first confirmed case was announced February 26, 2020 in the city of São Paulo. At the time of this article’s publication there were more than 600,000 confirmed cases and more than 36,000 deaths in Brazil (6,7).

Following the WHO declaration, the organization recommended the cancellation of elective surgeries in hospitals (5,8) due to the concern that elective procedures may contribute to the dissemination of COVID-19 and to optimize medical resources for emergency areas (9-11). Since then, safety protocols have been adopted for patients and health professionals to enable the continued execution of both elective and necessary surgical procedures during the pandemic (12-16). However, women’s needs for care due to gynecological disorders continue, as well as the need for special public health measures to avoid contagion during care.

### Medical care

Non-oncologic gynecological diagnoses are common and described as the main demand on women’s health care in reference centers. In the reproductive period, non-inflammatory and inflammatory diseases of the lower genital tract, such as abnormal uterine bleeding and pelvic inflammatory disease, respectively, are common. In the postmenopausal period, urogenital dysfunctions and breast diseases are prevalent (17). Protocols for obstetric care and maternal and child health care during the pandemic have been described (18,19), but there are few guidelines for women's health care in relation to gynecological disorders (18-22).

In recent publications, we discussed the importance of systematization and organization of work processes, prioritizing activities, and implementing clinically relevant algorithms in each specialty (12-16); these measures should be oriented to patient safety and guide decision-making for appropriate surgical treatment, both of which are appropriate concerns for the gynecological field.

Another concern during this period of the COVID-19 pandemic is the indefinite postponement of surgical treatment, which can aggravate the health and quality of life of women with hemorrhagic, pain, and/or genitourinary disorders (12-17).

A gynecologist’s decision is fundamental in the definition of elective procedures that may be postponed depending on the general and clinical status of the patient, the availability of access to clinical treatment in the Unified Health System, and the conditions and diagnoses to be elucidated that may or may not be expected (due to a delay in the time of diagnosis) for medical reasons. Thus, the American College of Surgeons proposed stratification of surgical cases according to the patient’s clinical condition and the severity of the disease as low, intermediate, or high severity. Stahel used the indications and waiting times to stratify the following for general surgical cases: emergency surgeries (<1h), urgent surgeries (<24h), elective urgent surgeries (<2 weeks), essential elective surgeries (<3 months), and non-essential elective surgeries (>3 months) (9-11). These guidelines could also be used for gynecological surgeries.

**Figure 1:**
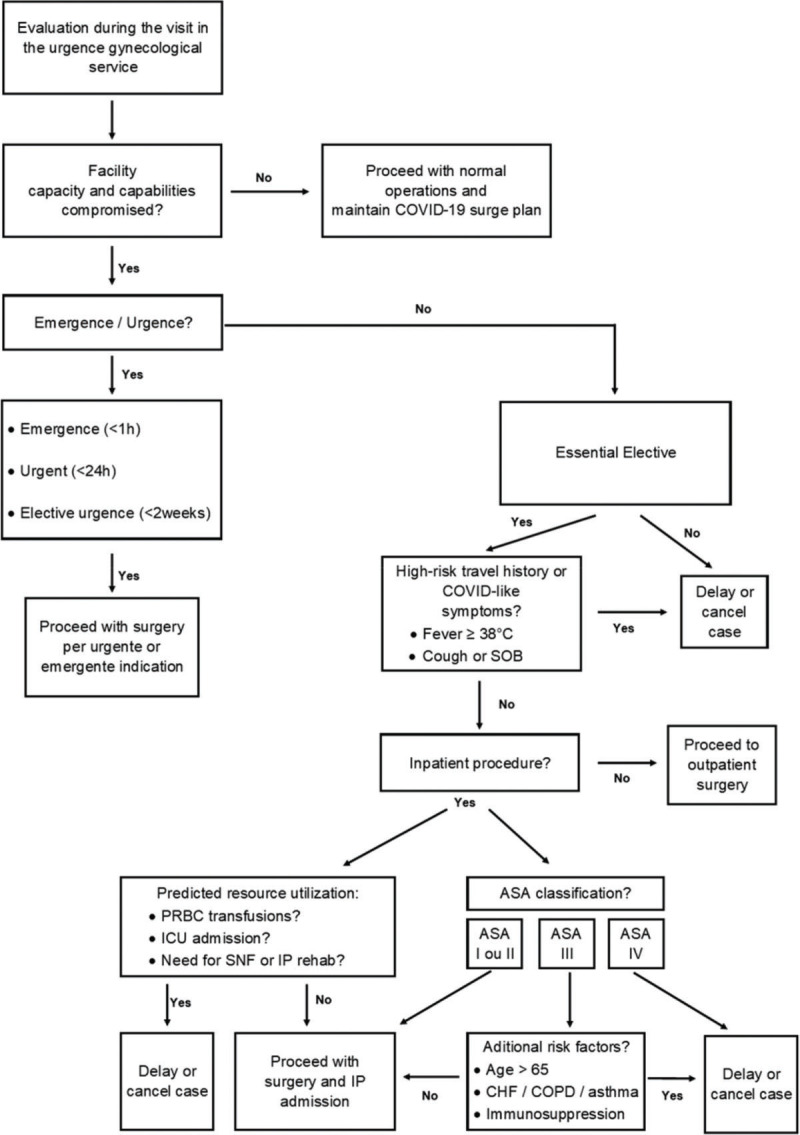
Proposed decision-making algorithm for risk-stratification of elective surgical procedures based on the underlying surgical indication and predicted resource utilization during the coronavirus disease (COVID-19) pandemic. Abbreviations: ASA, American Society of Anesthesiologists; CHF, chronic heart failure; COPD, chronic obstructive pulmonary disease; COVID, coronavirus disease; ICU, intensive care unit; IP, inpatient; PRBC, packed red blood cells; SNF, skilled nursing facility; SOB, shortness of breath. *Adapted from Stahel PF (10).

In this context, based on a recent publication regarding patient safety in elective surgeries (9-11), as well as on the law of access to treatment and laws related to women’s health care (23,24), we propose the inclusion of gynecological surgery cases, stratified as follows (Figure 1):

**Emergency (<1h):** Peritonitis by tubo-ovarian and/or pelvic abscess, necrotizing fasciitis in surgeries for pelvic and breast neoplasms;
**Urgent (<24h):** Postoperative infections, acute inflammatory abdomen (adnexal tortoise, myoma tortoise, ovarian cysts), hemorrhagic conditions (ovarian cysts);
**Elective urgent (<2 weeks):** Surgeries for neoplasms of the lower genital tract and breast previously diagnosed by pathological examination;
**Essential Elective (>2 to <3 months):** Hysteroscopy for abnormal uterine bleeding (unknowledge causes, suspected malignancy, and menopausal transition), postmenopausal bleeding (suspected malignancy), cervical conization or looped electro excision procedure (to exclude neoplasm in the lower genital tract);
**Non-essential/elective surgery:** Infertility procedures, family planning procedures (bilateral tubal ligation procedure).

A patient may obtain gynecological care from providers offering daily reception and/or urgent and emergency care.

It should be noted that provider locations may be far from those considered priority cases due to COVID-19. This should also be considered when determining the condition severity and surgical risk of each patient.

Furthermore, the estimated length of postoperative hospitalization should be abbreviated, and preference should be given to minimally invasive surgeries. The cost (possibility of resources) and surgical indication should always be reassessed if there is a need and/or expected risk for prolonged ventilation in the postoperative period (25-28).

There is no consensus in the literature regarding whether laparoscopy or laparotomy is superior under pandemic conditions. However, the principles of safety and testing whenever possible should be followed. In suspected or confirmed cases of COVID-19, the preferred route should be the one that produces the lowest aerial dispersion of viral particles. In urgent/emergency cases or in surgical cases with possible intestinal involvement, laparotomy would be preferred (25-28).

When evaluating surgical indications, a gynecologist’s decision is made individually. The analysis of a clinical case is based on guidelines in addition to the gynecologist’s experience.

In the acute phase of the COVID-19 outbreak, self-regulation has been observed; patients may voluntarily cancel scheduled elective consultations and procedures (9-11).

We must remember that reproductive planning is a right guaranteed by law. All contraceptive methods are considered safe for use, and eligibility criteria (use and safety) are maintained. Access to contraceptive methods may be compromised due to several factors, including lack of access to a prescription that must be administered by a health professional. Thus, behavioral and barrier methods should always be encouraged. Furthermore, health professionals should give preference to the maintenance of long-term contraceptive methods or those previously used by the patient. Long-term methods should be maintained, and the exchange time may be extended without prejudice to the patient’s reproductive planning (25-28).

The care of patients with non-surgical gynecological complaints should be postponed, and, when possible, these patients should be encouraged to make use of telemedicine services (9-11).

### Protection of gynecologists

Professionals in the operating room should be limited to the essential, and the use of complete footwear protection, waterproof aprons, surgical or N-95 masks, head protection, gloves, and eye protection (glasses or face shield) should be ensured. Movement in and out of the operating room should be limited to what is strictly necessary (25-28).

N-95 masks have been shown to be 95% effective in filtering particles larger than 300 nm. They should be effective in filtering SARS-CoV-2 particles range from 50 to 200 nm. Good-quality conventional surgical masks can provide protection similar to that of N95 masks under general-purpose conditions (25-28).

Many gynecological procedures can be performed using a locoregional block (e.g., spinal anesthesia, epidural). As such, we can often opt for this type of anesthesia and avoid the orotracheal intubation necessary for general anesthetic procedures to safeguard the anesthesiology team (25-28).

In addition to contagion by contact with surfaces and secretions due to manipulation of the patient, it is theoretically possible that aerosolization of viral particles through the use of cautery instruments and dissection (i.e., electrosurgical and ultrasonic scalpels) may be a source of transmission, especially in surgical times such as opening valves of trocars in endoscopic surgeries or extraction of parts, as in vaginal surgeries (25-28).

Despite the theoretical speculation for this type of transmission, one should be careful during these surgical times to avoid exposure of the team to viral aerosols. Therefore, when using electrosurgical or ultrasonic elements, a lower power should be used to reduce smoke/steam production. Providers should also maintain aspiration and perform dissection for shorter intervals. Vacuum cleaners with closed systems and ultra-small particulate matter filtration are indicated and may minimize this problem (25-28).

Procedures in cervical pathology, such as laser vaporization, conization, and high frequency surgery, usually produce smoke and vapors. Therefore, greater attention should be given to the protection of professionals, and the proper use of energy sources and smoke evacuation should be ensured to minimize contamination of the environment (25-28).

In laparoscopic/robotic surgeries, direct deflation should be avoided and the least possible intraabdominal pressure should be used (e.g. 10-12mmHg). Care should be taken during instrument exchanges and removal of surgical specimens (25-28).

In this context, it is also important to avoid the spreading of liquid and/or blood droplets during instrument manipulation (25-28).

In diagnostic hysteroscopy, contamination is theoretically possible via the use of distension means, especially gas. It is recommended to use liquid (saline) as a means of distension (25-28).

## CONCLUSION

In summary, non-surgical treatments should be used whenever possible to reduce the risk of horizontal transmission of SARS-CoV-2 to health professionals and the general population and, therefore, reduce the need for hospitalization. Patients should be evaluated for possible viral infection, and universal screening should be considered for all surgical candidates and patients undergoing surgical procedures. Urgent and emergency personnel should always be suspected of SARS-CoV-2 contamination, and appropriate safety procedures and equipment should be utilized by all health professionals.

## AUTHOR CONTRIBUTIONS

Soares-Júnior JM, Sorpreso ICE, Motta EV, Utiyama EM and Baracat EC conceived and planned the present idea. Soares-Júnior JM, Sorpreso ICE, Motta EV and Utiyama EM revised the literature and developed the theory manuscript. Soares-Júnior JM, Utiyama EM and Baracat EC took the lead in writing the manuscript. All of the authors provided critical feedback and helped shape the research, analysis and manuscript.
